# Open-source design of low-cost sensorised elastic actuators for collaborative prosthetics and orthotics

**DOI:** 10.1016/j.ohx.2024.e00564

**Published:** 2024-07-23

**Authors:** Filippo Sanfilippo, Martin Økter, Jørgen Dale, Hua Minh Tuan, Muhammad Hamza Zafar, Morten Ottestad

**Affiliations:** Department of Engineering Sciences, University of Agder (UiA), Jon Lilletuns vei 9, Grimstad, NO-4879, Norway

**Keywords:** Elastic joint, Prosthetics, Orthotics, Open-source, Cobots

## Abstract

Collaborative robots, or cobots, have become popular due to their ability to safely operate alongside humans in shared environments. These robots use compliant actuators as a key design element to prevent damage during unintended collisions. In prosthetic and orthotic applications, compliant actuators are crucial for ensuring user safety and comfort. However, most compliant cobots for these applications are excessively expensive and complex to construct. Our study introduces an innovative, cost-effective, and sensorised elastic actuator design tailored for prosthetics and orthotics. The design uses a modular approach and leverages 3D printing technology for rapid customisation, enabling efficient and affordable fabrication. Both hardware and software components are open-source, facilitating unrestricted access for students, researchers, and practitioners. Our design supports impedance and admittance control techniques, enhancing the system’s capabilities. Validation results show a standard deviation of 9.67 Nm between calculated and measured torque in impedance control and 0.2563 radians between calculated and measured angles in admittance control. This allows for improved adaptability to varying operational requirements in prosthetics and orthotics. By introducing this educational framework encompassing a low-cost, sensorised elastic actuator design, we aim to address the need for accessible solutions in the field of collaborative robotics for prosthetics and orthotics.

## Graphical abstract

**Figure:**
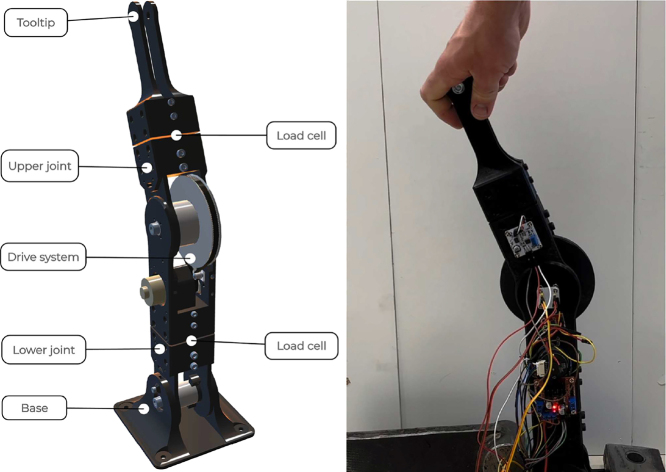

## Specifications table

**Table:** 

Hardware Name	Low-Cost Sensorised Elastic Actuator for Collaborative Prosthetics and Orthotics
Subject Area	Mechatronics, Biomedical, Robotics
Hardware Type	Sensorised Elastic Actuators based Cobot
Closest Commercial Analog	Passive, non-sensorised orthotics
Open Source License	MiT License
Cost of Hardware	∼100 US$
Source of File Repository	https://doi.org/10.17605/OSF.IO/4EUJQ

## 1 Hardware in context

Robotics has evolved as a fascinating and quickly developing area in recent decades, with a wide range of applications in a variety of sectors. The rise of collaborative robots, often known as cobots, which are meant to operate safely alongside humans or in shared environments, is one of the most significant advances in robotics. Because of their capacity to improve efficiency, productivity, and safety, cobots have grown in popularity in recent years, particularly in industrial settings.

Compliant actuators, which are designed to avoid harm in the case of an unintended collision, are commonly used in cobots. These actuators are critical for guaranteeing the safe and effective collaboration of robots and people, especially in environments where there is a potential risk of injury for humans or damage to equipment. In dynamic settings, elastic joints are preferable over rigid joints because they can absorb energy and lessen impact force in the case of a collision.

Despite their advantages, compliant cobots are frequently prohibitively expensive or difficult to build, limiting their accessibility and potential uses. This issue is particularly significant in the field of prosthetics and orthotics, where the usage of compliant actuators is critical for guaranteeing the safety, comfort, and natural movement of assistive devices.

Prosthetics and orthotics are medical disciplines that deal with the design, manufacture, and fitting of prosthetic limbs, braces, and other assistive equipment. Individuals with limb loss, limb difference, or musculoskeletal disorders use these tools to live more autonomous and functioning lives. Unfortunately, developing and building compliant actuators for prosthetic and orthotic devices is frequently a difficult and expensive endeavour, restricting their availability and affordability for many individuals.

**Fig. 1:**
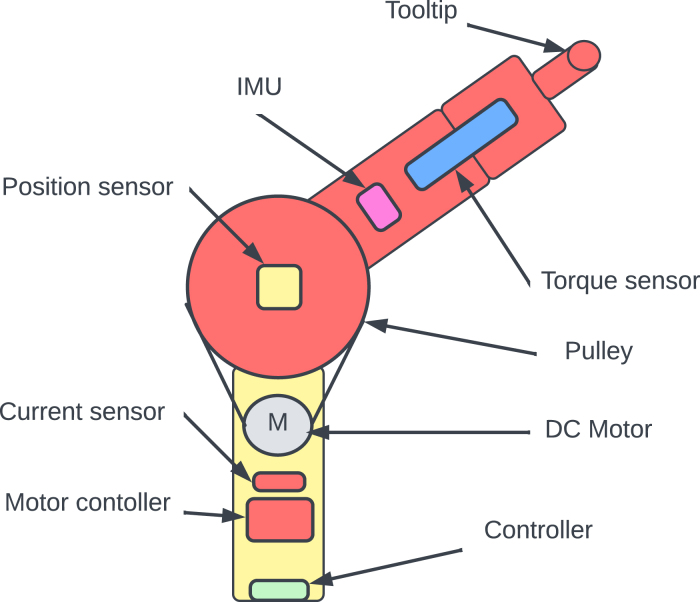
Hardware model of the proposed elastic arm.

To address this issue, this research presents an innovative low-cost sensorised elastic joint design for prostheses and orthotics, which is outlined in Fig. 1. This is introduced as an educational framework. The modular design allows for easy customisation utilising 3D printing technology, enabling for efficient and cost-effective manufacture. Both the hardware and software are open-source. Students, researchers and practitioners can fully access the project at OSF https://osf.io/4eujq/, along with several detailed diagrams, documentation and demo videos. The design also allows for the implementation of impedance and admittance control techniques, providing even greater versatility to the joint. The proposed design could potentially be used to produce more affordable and accessible prosthetic and orthotic devices.

There is a rising interest in the use of compliant and sensorised joints in the field of prosthetics and orthotics to increase user safety and comfort. Compared with their widely used rigid counterparts, soft actuators and robotic devices can provide a range of significant advantages; these include safe interaction, a range of complex motions, ease of fabrication, and resilience to a variety of environments [1]. In an effort to improve energy efficiency and minimise impact forces while walking, compliant actuators have been developed for prosthetic legs [2]. In the literature, the first powered ankle with a series elastic actuator (SEA) configured with a parallel spring to improve torque bandwidth in the transition from controlled dorsiflexion to powered-push-off was introduced in [3]. According to [4], the ability of the actuator to contribute energy during powered push-off improved the metabolic cost of walking. Since then numerous actuators have been designed. In [5], a powered knee prosthesis with small-scale, light-weight and affordable series elastic actuator was introduced and tested on one above knee amputee, walking on a treadmill. In [6] a novel robotic prosthetic knee with variable transmission mechanism that could vary transmission ratio while knee angle varies during ambulation activities was outlined. In [7], a new nonlinear series elastic actuator based on the conjugate cylindrical cam was proposed for the actuation of the prosthetic knee in level-ground walking. In [8], the development of an actuator for lower-extremity powered prosthesis that mimics the biological function of human knees and ankles was presented. The actuator is designed for maximum energy economy and has been demonstrated to fulfil the torque, angle, and velocity requirements for walking on flat ground and diverse terrain. The actuator’s performance is proven on a benchtop as well as on a human subject with a below-knee amputation. Despite the success in applying elastic actuators for prosthetic legs, the systems available are relatively expensive and complex.

Notwithstanding the success of the adoption of elastic actuators for prosthetic legs, the use of compliant actuators in prosthetic arms is limited because to the high torque and accuracy required [9]. To the best of our knowledge, a low-cost elastic actuator system featuring a modular design with integrated sensors for prosthetic and orthotic applications is still missing.

The contributions of this work are as follows:

• a novel, cost-effective, and sensorised elastic actuator design specifically tailored for prosthetics and orthotics is proposed.
• modular design that leverages the capabilities of 3D printing technology to enable rapid customisation, resulting in efficient and affordable fabrication processes.
• open-source hardware and software that facilitate unrestricted access for students, researchers, and practitioners.
• possibility of implementing impedance and admittance control techniques, which allows for enhanced adaptability to varying operational requirements in prosthetics and orthotics applications.

## 2 Hardware description

**Fig. 2:**
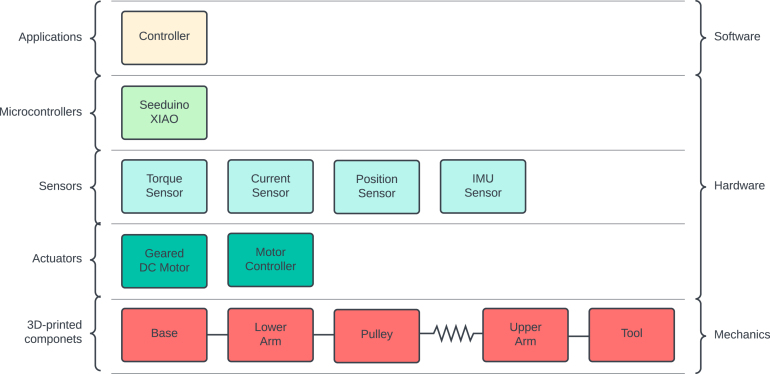
A comprehensive overview of the proposed system is presented as a block diagram, organised into the respective subsystems: mechanical, hardware, and software.

In this section, the mechanical overview of the proposed low-cost sensorised elastic actuator is depicted by highlighting the selected design principles, the mechanical design, the estimated production cost, the possible configurations and the adopted actuation system. A simple robotic arm is built for the sake of illustrating the design approach. Successively, the hardware overview is outlined. Later, the software description is also given. In Fig. 2, a full overview of the entire proposed system is given as a block diagram organised into the respective subsystems, i.e., mechanical, hardware, and software.

### 2.1 Mechanical description

#### 2.1.1 Design principles

The utilisation of 3D printing technology significantly contributes to the cost-effective strategy and accessibility of the robotic arm. As an additive manufacturing technique, it enables rapid prototyping and iterative design improvements, reducing both the time and resources spent on product development. By using affordable, widely accessible materials such as polylactic acid (PLA) or polyethylene terephthalate glycol (PETG), the production costs of the robotic actuator are significantly reduced. Additionally, 3D printing allows for a high degree of customisation and modularity of the actuator’s components, promoting the use of available, local resources and reducing the dependency on sub-contractors. This simplifies the production process, while encouraging a more collaborative development environment. As a result, the use of 3D printing not only lowers the overall cost of the product but also enhances its accessibility, fostering greater adoption and innovation in the field of robotics.

**Fig. 3:**
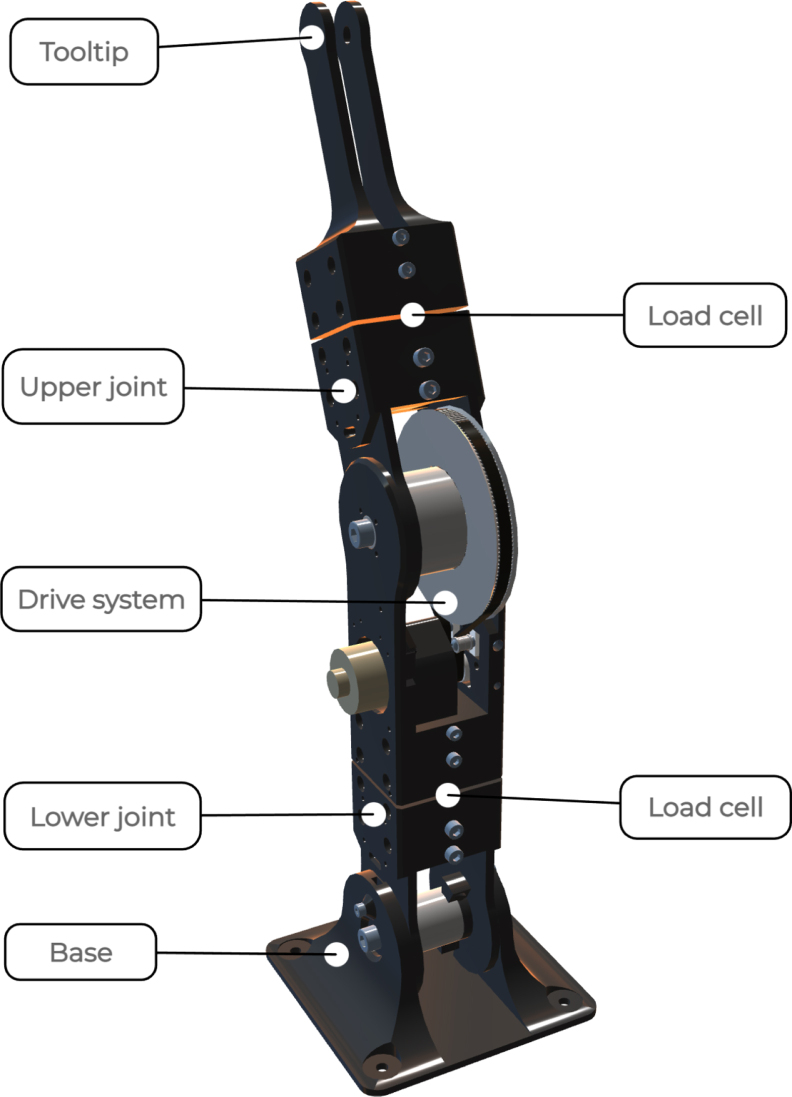
Full assembly of the proposed low-cost sensorised elastic joint design for prostheses and orthotics.

#### 2.1.2 Mechanical design

The mechanical construction consists of identically designed modules, which are shown in Fig. 3. The design includes a base, one or more joint modules, and a tooltip. Each degree of freedom (DoF) is obtained by connecting a lower joint, a drive system, and an upper joint. The lower joint consists of two parts joined together using a load cell [10]. This way the force across the joint can be measured. The drive system contains the motor, belt and pulleys, as well as the spring mechanism for elastic actuation. The upper joint features a mount for an additional load cell [10], connecting the joint to either a tooltip, or an additional drive system. This would render the upper joint identical to the lower joint, producing a serial robot with two driven joints.

**Fig. 4:**
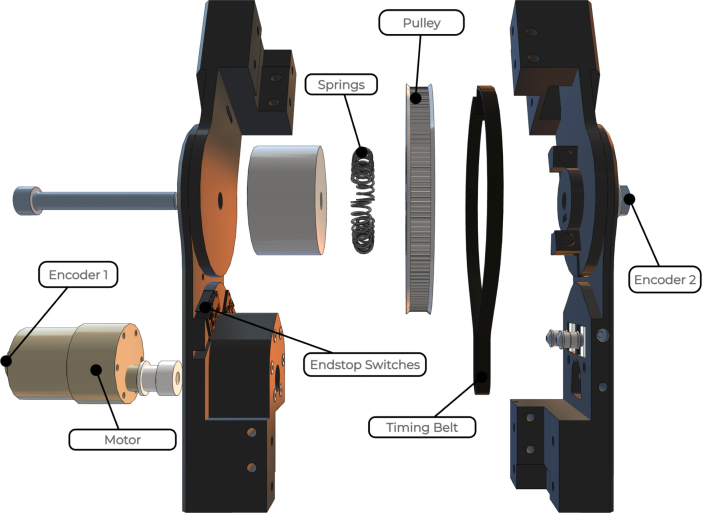
Exploded view of drive system.

The drive system is characterised by the parameters summarised in Table 1. An exploded view of the drive system is shown in Fig. 4.

**Table 1:** Parameters of each joint module.

Parameter	Value
Weight	∼500 g
Main width/depth	75 mm
Base width	138 mm
Length between joint axes	230 mm
Degrees of freedom	1
Max joint travel	$\pm 110^{\circ }$
Max continuous joint torque	3.0 N m (at 12 V)
Max joint speed with no load	77RPM (at 12 V)
Operating temperature (actuators)	−5 °C∼80 °C

#### 2.1.3 Estimated production cost

The estimated production cost for the joint module is of 100 USD including the following elements:

• 3D-printing cost;
• Cost of COTS mechanical parts (e.g., springs, nuts, bolts, bearings);
• Electrical components (e.g., microcontroller, sensors, actuator).

#### 2.1.4 Possible configurations

**Fig. 5:**
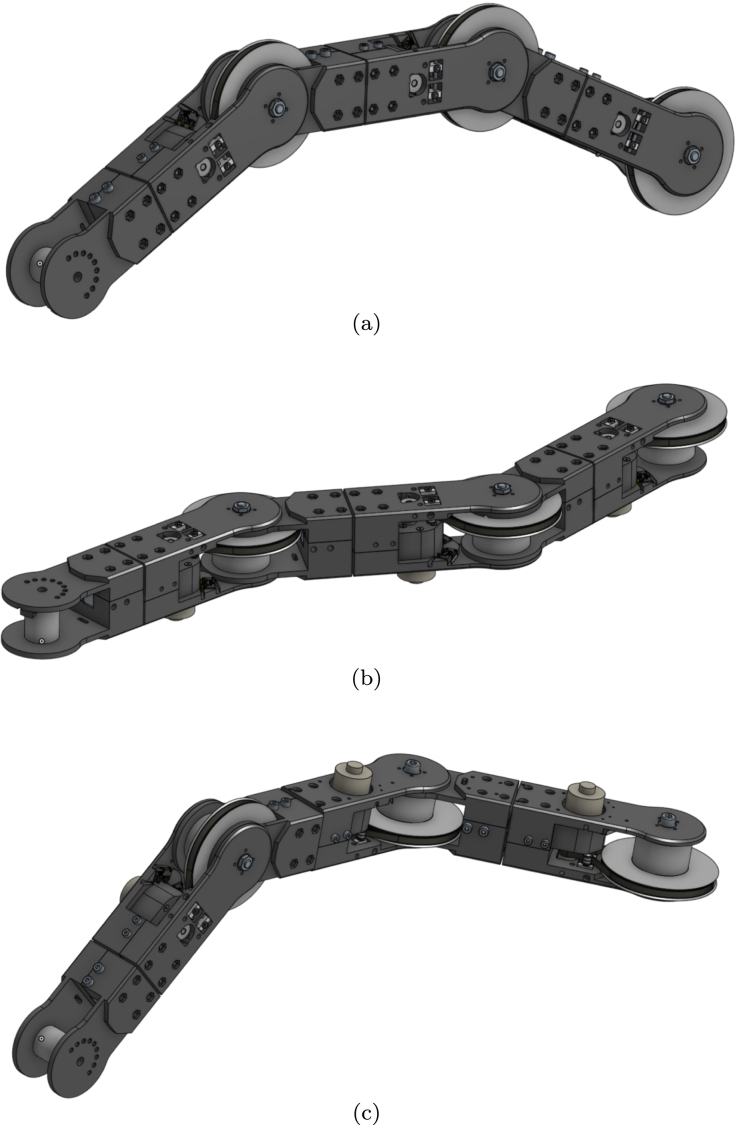
Different connections can be achieved: (a) pitch connection; (b) yaw connection; (c) pitch-yaw connection.

As shown in Fig. 5, the proposed mechanical design allows for realising different connections, such as pitch connection, yaw connection and pitch-yaw connection.

#### 2.1.5 Series elastic actuator (SEA)

**Fig. 6:**
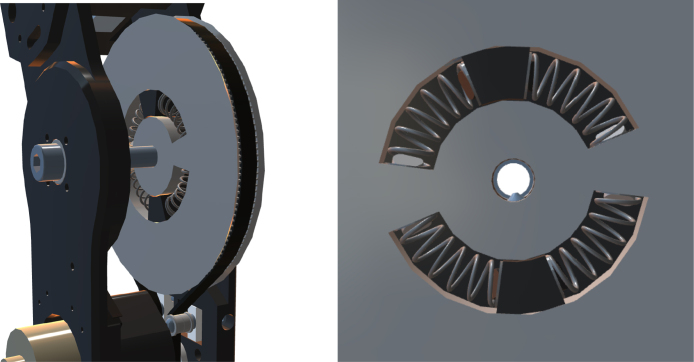
Series elastic actuator (SEA).

The actuator’s mechanical design is based on a thorough selection of essential components and necessary functionality for the final product. The fundamental objective is to incorporate elastic actuation principles, wherein the joint’s position in relation to the actuator is not fixed but allows for yielding instead of causing harm or damage. This goal is achieved by using a belt-driven motor system in conjunction with radial springs that link the secondary pulley to the joint, as shown in Fig. 6. To achieve force sensing capabilities, each joint section accommodates a load cell, and ensures that the forces are evenly distributed across it. The selected motor features an encoder on the motor shaft to track the position. However, due to the elastic nature of the actuator, the motor position and joint position are not necessarily linearly dependent. Therefore, a second encoder is fitted on the joint itself, providing absolute reading of the joint position.

### 2.2 Hardware description

#### 2.2.1 Microcontroller

In order to meet the stringent requirements for haptic control loop, a microcontroller with a high clock frequency is required. While microcontrollers with improved performance characteristics are more expensive, there is a need to find a low-cost alternative. The Arduino Due could be a valid option with 84 MHz [11], [12], [13], for comparison the original UNO has 16 MHz [14]. However, due to cost considerations, alternative possibilities were researched. Several ESP32 boards [15] were also considered as viable options. Throughout the prototyping process, both the Arduino Due and the Seeeduino XIAO were used as test platforms [16]. It was consistently noted that the Seeeduino XIAO outperformed the Arduino Due, particularly while delivering serial output data to the serial monitor. The threading approach adopted by the Seeeduino XIAO allows the retrieval of crucial data necessary for tuning and data gathering, all while keeping a tolerable loop time. Therefore, the Seeeduino XIAO processor was finally picked as the best solution. The Seeeduino XIAO chip is built on a M0+ chip with an ARM structure [17]. It has a clock frequency of 48 MHz, which is fast enough to achieve reliable haptic control. It is an affordable platform and have enough pinouts to support all necessary sensors required for control purposes.

#### 2.2.2 Motor

The selection of a motor is a strategic decision aimed at fulfilling the goals of cost-effectiveness, safety, and optimal actuation performance. Brushed DC motors are a widely available and cost-effective solution [18], making them an ideal choice for a low-cost educational and demonstrative tool. However, most DC motors run with a very high speed, unsuitable for slow, safe joint operation. Therefore, a motor with an integrated gearbox is selected. The integration of a gearbox allows arm to achieve the desired low-speed operation, while increasing the available torque. This not only ensures the safe actuation of the robotic joint, but also promotes better control and accuracy during movement. Even with the highest gear ratio available for the gearbox, the speed of the joint would still be excessive, so additional gearing is added through a belt-pulley system connecting the motor shaft to the joint, as shown in Fig. 4.

#### 2.2.3 Sensors

Four sensors are seamlessly integrated to enable comprehensive implementation of the haptic control loop. These sensors encompass both position and force feedback measurements, providing feedback from the load as well as the actuator. To accurately determine the position of the load, an AS5600 magnetic encoder [19] of suitable type is employed. The utilised sensor has a 12-bit output resolution for a complete rotation and provides stable and high-quality feedback in the form of an inter-integrated circuit (I2C) signal [20]. In the outer section of the arm, a load cell is implemented for the force feedback. The signals are routed through an instrument amplifier and provide an analogue 5 V signal. On the actuator side, a current sensor (ACS723) [21] is used to measure the current provided to the motor. This measurement can be used to approximate the force provided by the motor. For position of the actuator, the integrated hall sensor on the motor shaft is used.

### 2.3 Software description

To facilitate the control of the haptic arm, a series of classes are available, each containing key methods for distinct functionalities. Three low-level classes are designed to handle sensor feedback, actuator control, and proportional–integral–derivative (PID) [22] adjustments. Additionally, the “HapticArm” class is developed to encapsulate high-level control structures. The programming language chosen for this project is C++, primarily due to its simplicity of implementation on microcontrollers, lightweight nature, and adaptability. When implemented on an Arduino-based microcontroller, the main control file adheres to the Arduino coding style and is assigned the “.ino” extension.

**Fig. 7:**
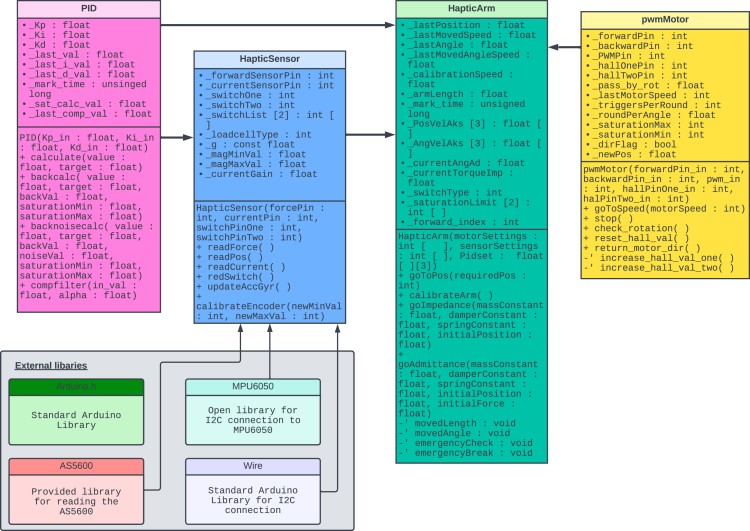
UML Class diagram of the control structure.

In Fig. 7, a Unified Modelling Language (UML) Class diagram is presented illustrating the proposed control program for the haptic arm. As the chosen controller is a Arduino, the standard library “Arduino.h” is utilised. This library contains the necessary build instructions for writing and reading pins, and therefore it is included in every classes. In addition to the standard library, three pre-existing libraries were used. The “Wire.h” library is a standard Arduino library for I2C communication. Moreover, the “AS5600.h” library is used for retrieving data from the AS5600 magnetic sensor, and the “MPU6050.h” library is adopted for retrieving data from the MPU6050 accelerometer.

The “HapticSensor” class makes use of the three pre-existing libraries to maximise their functionality. Four of the five sensors embedded into the haptic arm platform are implemented as methods in this class. Notably, the hall encoder for motor position sensing deviates from this pattern and is instead implemented within the “pwmMotor” class. The “HapticSensor” library consist of five methods (“readForce”, “readPos”, “readCurrent”, “readSwitch”, “calibrateEncoder”) in addition to the constructor. Four are the methods for returning sensor data, and the last one is for configuration of the end positions of the arm for the position sensor. In addition, a method for retrieving the accelerometer data using the “MPU6050.h” library is available.

The “pwmMotor” class has a number of distinct methods for controlling a PWM motor using an H-bridge type motor controller and data gathering from an attached hall encoder. The class consist of five public methods (“goToSpeed”, “stop”, “check_rotation”, “reset_hall_val”, “return_motor_dir”) in addition to two private ones and the constructor. The two private methods serve the purpose of gathering data as interrupts from the hall encoder and are defined as “static void” due to the necessity of this data type for methods interacting with interrupt variables. This requirement also accounts for the “static void” data type assigned to the “reset_hall_val()” method.

The PID class is intended to simplify the creation of PID loops by exploiting class object characteristics to allow users to easily create several loops with different PID values based on the same class. The back-calculation technique described by da Silva in [23] is used for the mathematical modelling of the anti-windup configuration. In addition to the constructor, the PID class has four public methods. One method allows for the creation of a typical PID loop using $K_{p}$, $K_{i}$, and $K_{d}$ parameters, while another method allows for the use of a PID controller with back-calculation integration as an anti-wind-up mechanism. The most advanced integrated method, “backnoisecalc”, includes a noise filter for the derivative term. A complementary filter, which acts as a low-pass filter on the sensor data, is also included. The PID method uses the same set of settings and can be tuned while running if required.

**Fig. 8:**
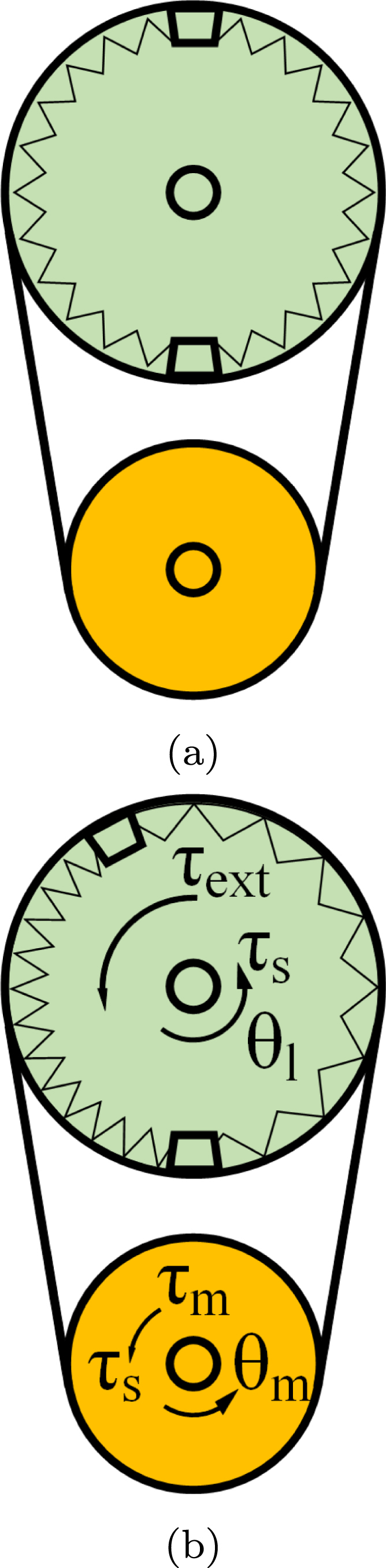
Elastic joint system: (a) without external force/torque; (b) deformed by external force/torque action.

### 2.4 Mathematical model and control

#### 2.4.1 Mathematical model

Fig. 8 illustrates the schematic diagrams of the elastic actuator. The gear ratio is $N=N_{l}/N_{m}>1$, where $N_{l}$ and $N_{m}$ are the number of teeth for the load and the motor gear, respectively. The torques that affect the system include the motor torque ($\tau_{m}$), the spring reaction torque ($\tau_{s}$), and the external torque ($\tau_{ext}$). When there is no external force/torque, the elastic joint is in its equilibrium position, as shown in Fig. 8(a). When the elastic joint is influenced by external action, illustrated in Fig. 8(b), the springs inside the joints are deformed, therefore creating elastic forces. The disturbances of the motor and the load is denoted as $d_{m}$ and $d_{l}$, respectively. Denoting the motor angular position as θ, the load angular position as q, the rotor inertia as $J_{m}$, motor damping coefficient as $D_{m}$, the stiffness coefficient of the spring as $K_{m}$, the spring damping coefficient as $D_{s}$, the load inertia as $J_{l}$, and the load damping coefficient as $D_{l}$. As elaborated in [24], [25] the mathematical model of the elastic joint can be written as:

(1) $$d_{m}+\tau_{m}-N^{-1}\tau_{s}=J_{m}\overset{¨}{\theta }+D_{m}\overset{˙}{\theta },$$

(2) $$\tau_{s}=K_{s}\left(N^{-1}\theta -q\right)+D_{s}\left(N^{-1}\overset{˙}{\theta }-\overset{˙}{q}\right),$$

(3) $$d_{l}+\tau_{s}+\tau_{ext}=J_{l}\overset{¨}{q}+D_{l}\overset{˙}{q}.$$

The relationship between the motor torque, the spring torque and the motor angular position on the motor-side is shown in Eq. (1). The elastic torque is shown in Eq. (2). Finally, the relationship between the spring torque, the external torque and the load angular position on the load-side is shown in Eq. (3).

#### 2.4.2 Control

The “HapticArm” class integrates the lower-level control classes of the haptic arm, aiming to exert advanced control theory for arm manipulation. In addition to the constructor, the library includes four public (goToPos, calibrateArm, goImpedance, goAdmittance) and four private methods (movedLenght, movedAngle, emergencyCheck, emegencyBreak). The two private methods use numerical mathematical techniques to calculate displacement, velocity, and acceleration based on position feedback. The “moved length” method returns the length of the tooltip’s displacement, whereas the “moved angle” method returns the angular displacement in the joint space. The last two methods act as emergency protocols, analysing available data to verify the arm is behaving normally and initiating the requisite stop routines if any irregularities are identified.

A key method named “calibrateArm” is provided within the class. This method is critical in guaranteeing the effective operation of emergency protocols. It is critical to carry out this procedure before proceeding with the control measures. When invoked, the method causes the motor to travel gradually in a certain direction until one of the end switches is triggered. Each switch is associated with the corresponding side of the arm, and when engaged, the motor reverses its direction until the other switch is activated. The angles obtained from the magnetic encoder, which correspond to the end-stops, are displayed and stored for subsequent utilisation in a mapping function to accurately calculate the corresponding angles in degrees. If the same arm is engaged multiple times, these values can be saved as default values, allowing for the exclusion of this method during subsequent testing to expedite the process.

Furthermore, an additional method named “goToPosition” is provided. This method belongs to the class and works as a basic PID loop, exploiting the PID library’s features. The “goToPosition” method takes a target angle as input and adjusts the arm’s angle by regulating the motor using the given PID settings. If the arm is to be tested after assembly or control tested, this method may provide useful information. Furthermore, this method is used as an inner loop for admittance control. A well tuned PID is indeed a necessary prerequisite for effectively achieving admittance control.

**Fig. 9:**
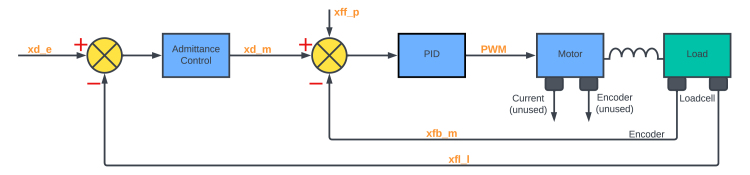
Block diagrams of the admittance control structure.

**Fig. 10:**
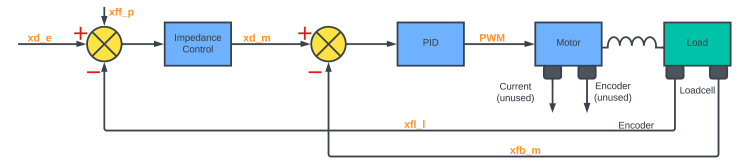
Block diagrams of the impedance control structure.

#### 2.4.3 Admittance

Admittance control is a type of control method that allows a robot to follow external forces by calculating the desired position, velocity, and sometimes acceleration using the equation of motion of a virtual object in response to external forces [26]. In the admittance control approach, a position controller is placed in the innermost loop. Firstly, the position correction quantity $x_{new}$ is calculated based on the desired impedance model [27]:

(4) $$\tau =M\left(\overset{¨}{x}-{\overset{¨}{x}}_{d}\right)+D\left(\overset{˙}{x}-{\overset{˙}{x}}_{d}\right)+K\left(x-x_{d}\right)$$

(5) $$\Rightarrow x_{new}=\frac{\tau -M\overset{¨}{e}-D\overset{˙}{e}-Kx}{K}$$

where τ is the interaction torque, x is the feedback position, $x_{d}$ is the desired trajectory, M is the desired mass coefficient, D is the desired damping coefficient, K is the desired stiffness, and $e=x-x_{d}$ is the error between the actual position and the desired position. The $x_{new}$ quantity is then used as the input for a PID position controller, as shown in Fig. 9 for admittance control, and in Fig. 10 for impedance control.

The “goAdmittance” method enables the user to control the arm using admittance control, which means reading the input force and controlling the position of the arm. The system will act as a spring damper system centred around the given initial position value. The private method “movedLenght” is used to find the moved length since last iteration. The length is based on the given length of the arm and uses trigonometry. It is an estimate based on numerical iteration of a curve divided into triangles, and the frequency of the code are important for the validation of the method.

The actual applied force are read by the use of the sensor object and the corresponding offset length are calculated using Eq. (5). The calculated length is obtained by retrieving the moved angle, from which the moved tangent of the tooltip is calculated. Finally, the required angle is sent to the “goToPosition” method used for the inner loop. The emergency test is embedded in the “goToPosition” method.

#### 2.4.4 Impedance

Impedance control is an approach to dynamic control relating force and position [26]. In the impedance control approach, a force/torque controller is placed in the innermost loop. Firstly, the desired torque is calculated directly from Eq. (4). Then it is fed into a PID torque controller, as shown in Fig. 10.

The “goImpedance” method enables the user to control the arm using impedance control, which means reading the displacement of the arm and adjusting the output torque of the motor. The private method “movedAngle” is used to find the angular displacement of the arm and the current is read by using the sensor object. The displacement values are then multiplied with the mass, spring, damper values, which are given by the user as inputs for the method. Eq. (4) is directly utilised for these calculations. The resulting torque, in conjunction with the measured current, is employed to compute a new velocity, which is then fed back to the motor. An embedded emergency test is incorporated within the method to ensure safety.

## 3 Design summary

**Fig. 11:**
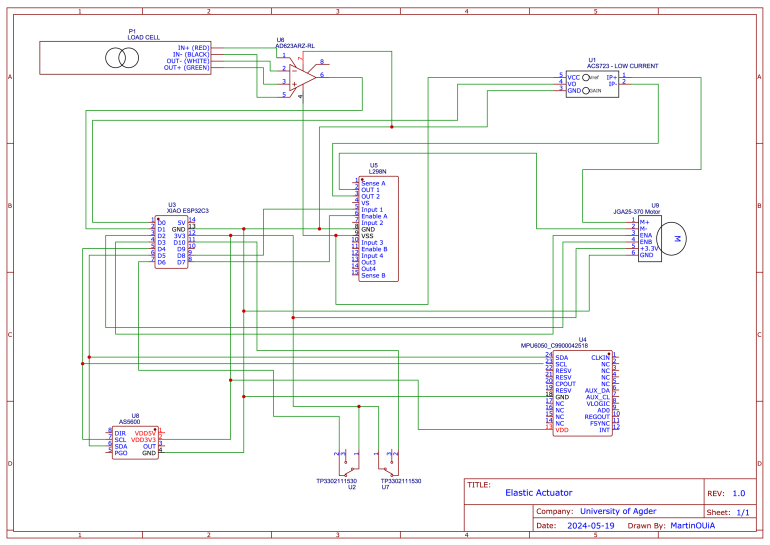
Schematic of the elastic actuator.

The design file summary of the low-cost sensorised elastic actuator for collaborative prosthetics and orthotics is presented in Table 2,

A complete list of all the adopted sensors can be found in Table 3. In Fig. 11, a complete schematic for the connection of the arm is presented.

**Table 2:** File summary of the needed parts, software and corresponding links to repository.

Design file name	File type	Open source licence	Location of the file
**Software**			
AS5600	.h/.cpp C++ library	MIT	https://osf.io/2we96/
hapticarm	.h/.cpp C++ library	MIT	https://osf.io/2we96/
hapticsensor	.h/.cpp C++ library	MIT	https://osf.io/2we96/
I2Cdev	.h/.cpp C++ library	MIT	https://osf.io/w2xz5
MPU6050	.h/.cpp C++ library	MIT	https://osf.io/y6h4u
pid	.h/.cpp C++ library	MIT	https://osf.io/j2czn
pwmLibrary	.ion Arduino file	MIT	https://osf.io/gq4v7
pwmmotor	.h/.cpp C++ library	MIT	https://osf.io/wx5dh
testCode	.ion Arduino file	MIT	https://osf.io/wx5dh
**Hardware**			
Schematic_ElasticActuator	.pdf Schematic	MIT	https://osf.io/72p5n
**Mechanics**			
Base	.stl, object file	MIT	https://osf.io/ysg5t
BeltWedge	.stl, object file	MIT	https://osf.io/se5dv
IdlerCart	.stl, object file	MIT	https://osf.io/y2rqj
Joiunt_L	.stl, object file	MIT	https://osf.io/cyn93
Joint_S	.stl, object file	MIT	https://osf.io/exzqj
LowerLeft	.stl, object file	MIT	https://osf.io/9snxd
LowerRight	.stl, object file	MIT	https://osf.io/ysg5t
MagnetHolder	.stl, object file	MIT	https://osf.io/qyv75
Pully160	.stl, object file	MIT	https://osf.io/qyv75
TooltipLeft	.stl, object file	MIT	https://osf.io/qyv75
TooltipRight	.stl, object file	MIT	https://osf.io/qyv75
TolltipSpacer	.stl, object file	MIT	https://osf.io/ymqdx
UpperLeft	.stl, object file	MIT	https://osf.io/9y52k
Upper Right	.stl, object file	MIT	https://osf.io/25g4q

**Table 3:** Complete list of all the adopted sensors.

Component	Qty.	Comment
AD5600	1	Magnetic encoder
Load Cell (5 kg)	2	Corresponding instrument amplifier needed
ACS723	1	Current sensor
MPU6050	1	Accelerometer

## 4 Bill of materials summary

The bill of materials (BOM) is shown in Table 4.

**Table 4:** Complete BOM of the mechanical hardware.

No.	Name	Serial no.	Qty.	Prise per item	Link
1	Geared motor	GM37–520	1	15.27$	aliexpress.com/item/32846235423.html
2	Motor controller	L298N	1	0.30$	aliexpress.com/i/32800756190.html
3	Magnetic encoder	AS5600	1	6.00$	aliexpress.com/item/1005006399935661.html
4	Load cell 5 kg	–	1(2)	0.41$	aliexpress.com/item/4000781493675.html
5	Load cell amplifier	–	1	0.00$	Included with load cell
6	Current sensor	ACS723	1	17.5$	digikey.com/en/products/detail/sparkfun-electronics/SEN-14544/9452026
7	Microswitch	D2F	2	4.00$	https://tinyurl.com/yc4hcbx4
8	Pulley 20t 6 mm ID	–	1	5.00$	elefun.no/p/prod.aspx?v=52054
9	Bearing 8ID 16OD	–	2	14.00$	no.rs-online.com/web/p/ball-bearings/2346922
10	Bearing flanged 3ID	–	4	8.00$	no.rs-online.com/web/p/ball-bearings/6126007
11	GT2 timing belt 6 mm	GT2	320 mm	3.00$	elefun.no/p/prod.aspx?v=50262
12	Springs 7ID 10OD 50 mm	C03600511380M	4	32.00$	fjaer.net/c03600511380m
13	Jumper cable 40 cm	–	–	3.44$	aliexpress.com/item/1005006148662373.html
14	MPU6050	–	1	20.00$	https://tinyurl.com/26tmamct
15	Seeeduion XIAO	–	1	7.00$	https://tinyurl.com/3jxcvts6
16	Hex countersunk head screw	M3 × 6	6	1.00$	no.rs-online.com/web/p/socket-screws/3044788
17	Hex button head screw	M3 × 22	2	1.00$	no.rs-online.com/web/p/socket-screws/4838196
18	Hex socket screw	M4 × 16	2	1.00$	no.rs-online.com/web/p/socket-screws/4838225
19	Hex socket screw	M4 × 20	1	0.50$	no.rs-online.com/web/p/socket-screws/1871279
20	Hex socket screw	M4 × 40	20	25.00$	no.rs-online.com/web/p/socket-screws/0293347
21	Hex socket screw	M5 × 40	4	2.00$	no.rs-online.com/web/p/socket-screws/4680032
22	Hex nut	M3	2	0.20$	no.rs-online.com/web/p/hex-nuts/0189563
23	Hex nut	M4	18	0.50$	no.rs-online.com/web/p/hex-nuts/0525896
24	Washer	M3 × 0.8	4	0.20$	no.rs-online.com/web/p/washers/4830710
25	Base	–	1	8.00$	3D-print
26	Right-Lower	–	2	5.00$	3D-print
27	Left-Lower	–	2	5.00$	3D-print
28	Right-Upper	–	1	5.00$3D-print	
29	Left-Upper	–	1	5.00$3D-print	
30	120t pulley	–	1	1.00$3D-print	
31	Space cylinder	–	1	0.50$	3D-print
32	Idler cart	–	2	0.01$	3D-print

## 5 Build instructions

**Fig. 12:**
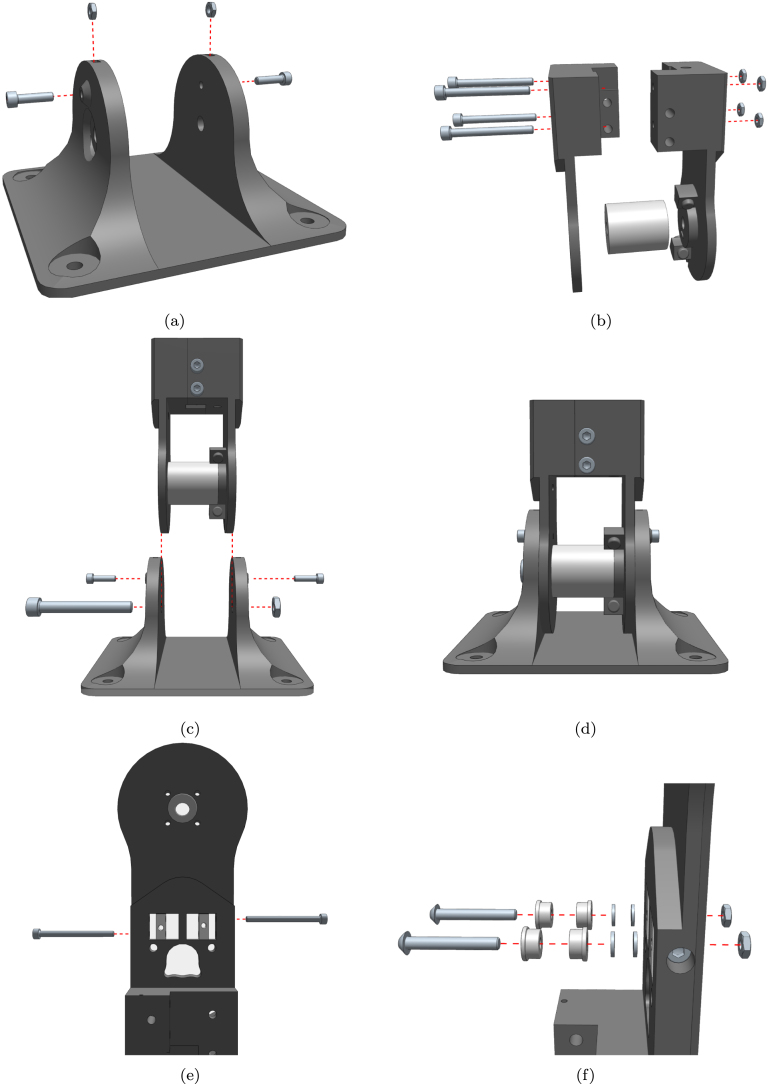

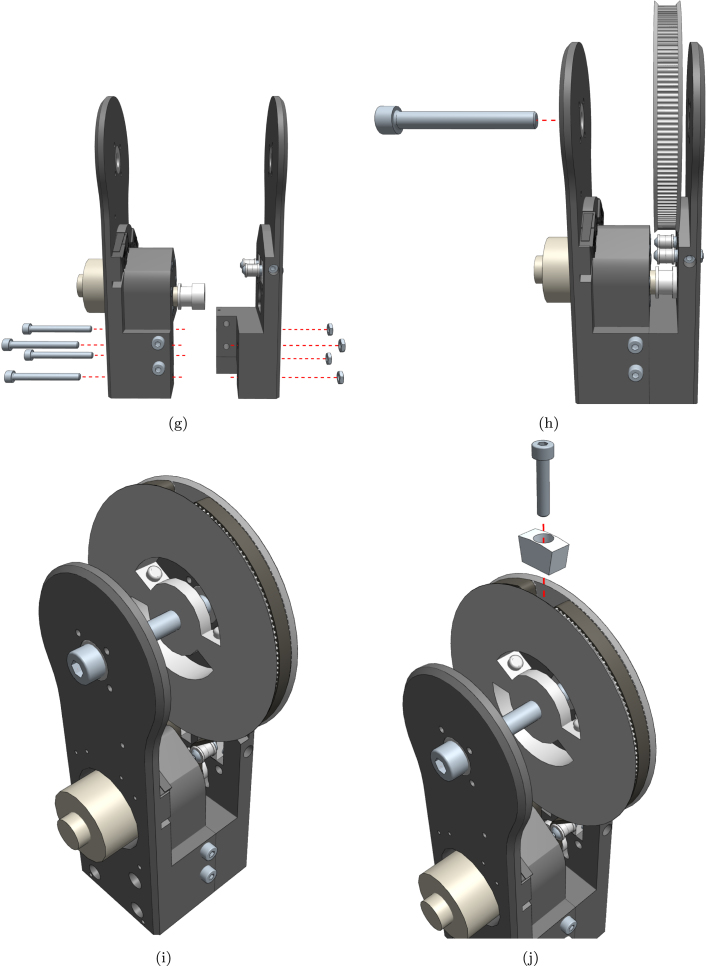

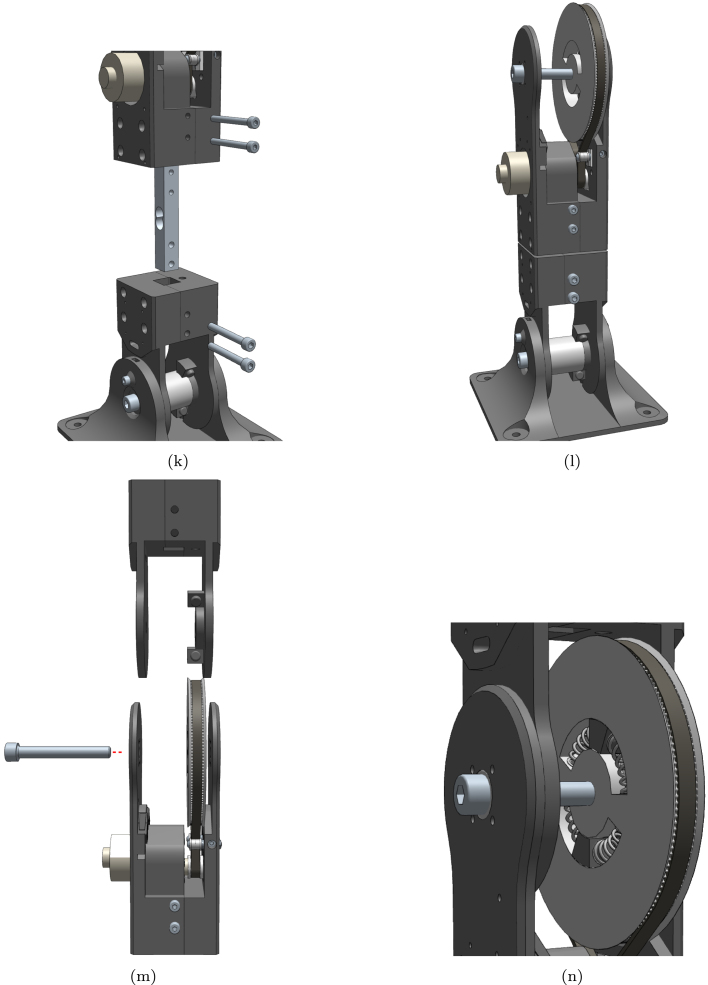

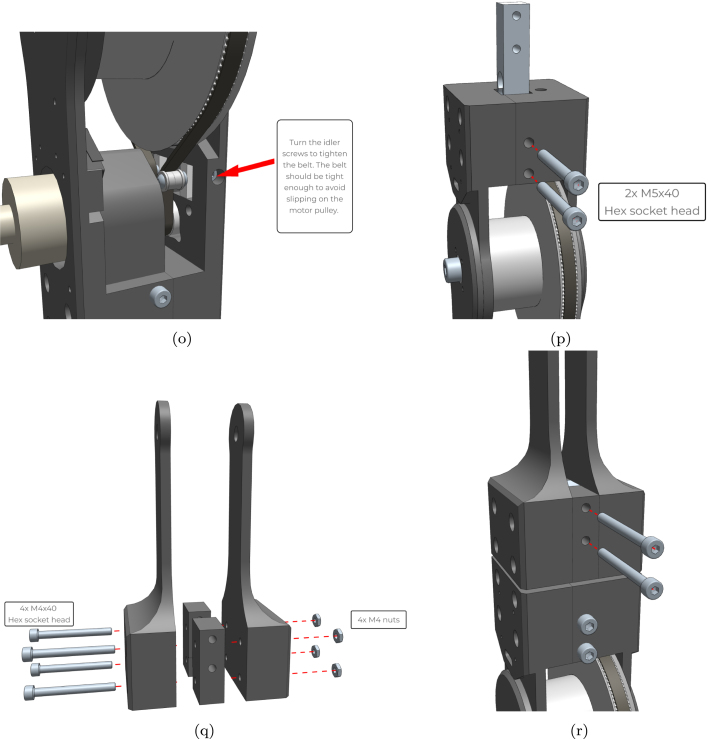
Detailed build instructions: (a) Start with the base. Insert the nuts from above and bolts from the side. Do not turn the bolts all the way until the next stage is mounted; (b) Assemble the lower joint using 4 bolts and nuts; (c) Mount the lower joint to the base using the centre locking bolts; (d) Lower section complete; (e) Install the idler carriers; (f) Install the idlers; (g) Mount the motor and assemble the upper joint; (h) Insert the large pulley and secure it temporarily with the centre bolt; (i) Thread the timing belt around the motor pulley, on the inner side of the idlers and around the large pulley. The ends should meet at the hole on top of the pulley; (j) Secure the belt in place using the wedge and screw; (k) Mount the upper joint to the lower joint and base using a load cell or a dummy; (l) Completed first joint; (m) Remove the centre bolt to insert another lower joint, and reinsert the bolt; (n) Insert springs in the cavities between the pulley and the lower joint and add the spacer by briefly removing the bolt; (o) Turn the idler screws to tighten the belt; (p) Secure a load cell to the new lower joint using the two M5 screws; (q) Assemble the toolhead; (r) Secure the toolhead to the load cell using the two M5 screws.

The assembly process consists of the following steps:

1. Start with the base. Insert the nuts from above and bolts from the side. Do not tighten the bolts completely until the next stage is mounted. (Fig. 12-a)
2. Assemble the lower joint using 4 bolts and nuts. (Fig. 12-b)
3. Mount the lower joint to the base using the centre locking bolts. (Fig. 12-c)
4. Install the idler carriers. (Fig. 12-e)
5. Install the idlers. (Fig. 12-f)
6. Mount the motor with pulley and assemble the upper joint similarly to the lower joint. (Fig. 12-g)
7. Insert the large pulley and temporarily secure it with the centre bolt. (Fig. 12-h)
8. Thread the timing belt around the motor pulley, on the inner side of the idlers, and around the large pulley. Ensure that the ends meet at the hole on top of the pulley. (Fig. 12-i)
9. Secure the belt in place using the wedge and screw. (Fig. 12-j)
10. Mount the upper joint to the lower joint and base using a load cell or a dummy (this load cell is only structural). (Fig. 12-k)
11. Completed first joint assembly. (Fig. 12-l)
12. Remove the centre bolt to insert another lower joint, and reinsert the bolt. (Fig. 12-m)
13. Insert springs in the cavities between the pulley and the lower joint, and add the spacer by briefly removing the bolt. (Fig. 12-n)
14. Turn the idler screws to tighten the belt sufficiently to prevent slipping on the motor pulley. (Fig. 12-o)
15. Secure a load cell to the new lower joint using the two M5 screws. (Fig. 12-p)
16. Assemble the tool head similarly to the other joints. (Fig. 12-q)
17. Secure the tool head to the load cell using the two M5 screws. (Fig. 12-r)

Refer to Fig. 12 for visual representations of each assembly step.

## 6 Operation instructions

### 6.1 Installation

After the meticulous completion of the mechanical assembly and the appropriate mounting of electronics, as detailed in Section 5, the next step involves connecting your chosen microcontroller to a computer. To program the microcontroller, an Integrated Development Environment (IDE) specifically designed for this purpose is required. The Arduino IDE serves as an excellent choice and can be easily obtained by downloading it from https://www.arduino.cc/en/software. Upon installing the chosen IDE, it is essential to integrate the Seeeduino board programming package. To accomplish this, access the Preferences section within the IDE and paste the following URL: http://files.seeedstudio.com/arduino/package_seeeduino_boards_index.json into the designated field labelled “Additional board manager URLs”. Once this step is completed, the Seeeduino package becomes accessible for installation via the board manager functionality within the IDE interface.

### 6.2 Test code

Begin by initiating the process with the cloning of this repository onto your computer, see Specifications Table. Navigate to the “Software\testCode” folder within the repository and proceed to open the “testCode.ino” file using the Arduino IDE. Upon ensuring the microcontroller’s connection, verify its availability by locating the corresponding port within the drop-down menu situated at the top section of the IDE window.

Next, designate the preferred control method by uncommenting the line that corresponds to the desired selection. For both the *goImpedance* and *goAdmittance* functions, specify the three initial arguments: mass, spring, and damper coefficients tailored to your system. In the case of impedance, the last argument, indicating the initial position, is optional. If left unspecified, the default position of 125 degrees, signifying the middle position, is assumed.

For the admittance function, the last two arguments are also optional. The default initial position remains at the middle (125 degrees = 2.18166 rad), while the initial force is set to 0, symbolising the system’s force sensitivity. Alternatively, setting the initial position to −1 will prompt the arm to maintain its current position, simulating a standard “teach the robot” mode.

The *goPos* function serves the purpose of basic position control within the system. Primarily utilised internally within the *goAdmittance* function, it is crucial to meticulously fine-tune this function before progressing to more advanced control methodologies. This step ensures optimal performance and seamless integration when transitioning towards the utilisation of more sophisticated control methods.

### 6.3 Test code explanation

This code establishes a foundational setup for testing the predefined control functions. Initially, fundamental parameters are configured, and the “hapticArm” class is employed to instantiate an “Arm” object. Should there be any modifications in the ports within the electronic setup or a necessity for adjustments in the PID controller, these values are adjustable to align with your specific preferences and requirements.

**Figure:**
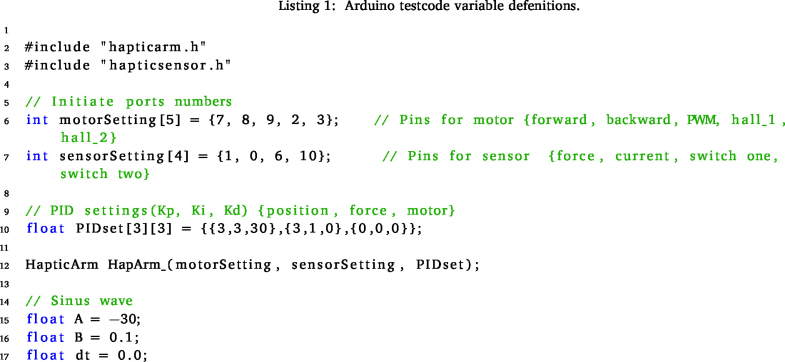

**Figure:**
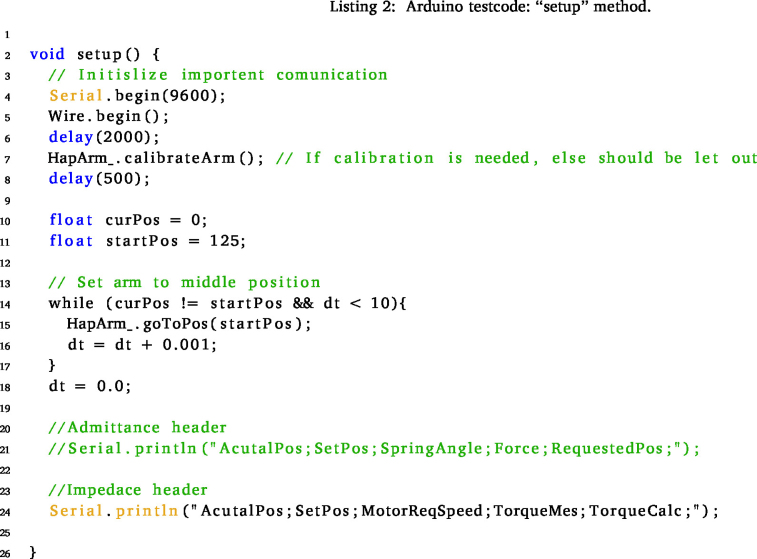

**Figure:**
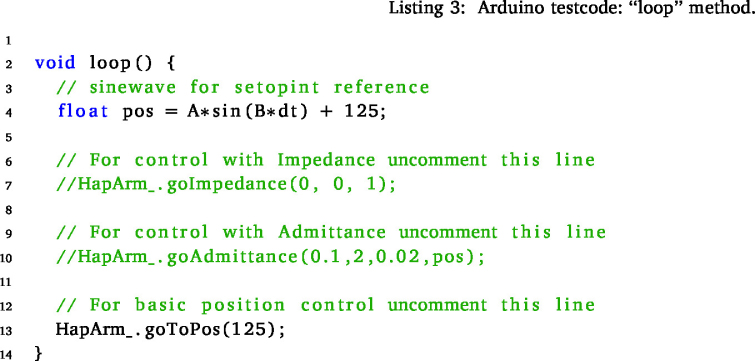

Within every Arduino code, two essential functions are present: the “setup” and “loop” functions. The “setup” function serves the purpose of configuring parameters and establishing input–output settings.

In this test code, the initial segment of the “setup” function involves the setup of a serial port, enabling communication with the computer for feedback purposes. Additionally, the I2C communication is executed using the “Wire.begin()” method.

The calibration sequence, integral to the “HapticArm” class, is pivotal. This actuates the arm to set the correct end switches and set the encoder values for the end point. This calibration is critical as these values are directly mapped to the corresponding arm angles, serving as a crucial metric for positioning. Moreover, the encoder limit values are displayed in the serial monitor. If these values wrap around 0, indicating an encoder issue, rotating the encoder chip by 90 degrees (1.5708 rad) is recommended, as the wrapping of encoder values has yet to be implemented.

A loop is employed to orient the arm in an upright position, optimising the system for smoother actuation upon initiation. This loop concludes after approximately 10 s. Lastly, a header providing an explanation for the admittance or impedance control function output is available for display in the serial monitor. Uncommenting this section can be done as necessary for clarity and understanding during operation.

The “loop” function operates continuously as long as the microcontroller remains powered. Within this function, the specified control function is executed for the “Arm” object.

**Fig. 13:**
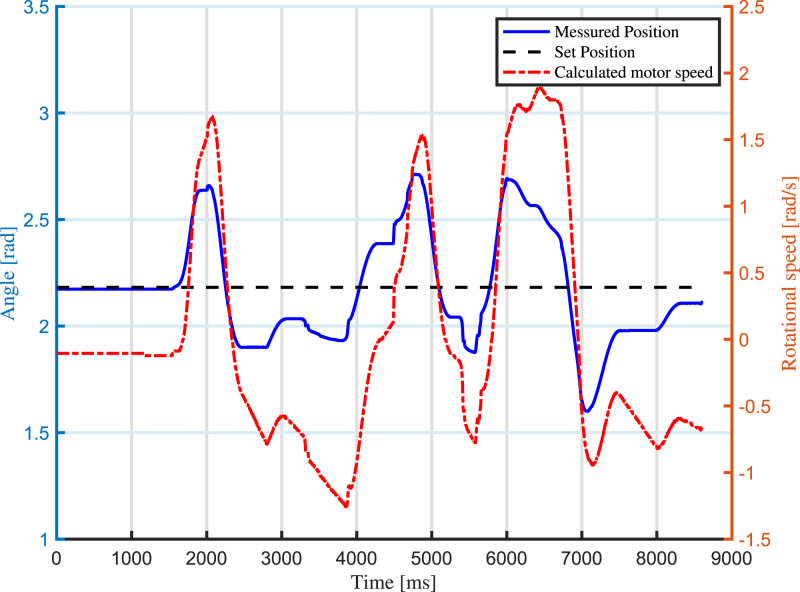
Angle position of the arm during the impedance control use case.

The initial line establishes a sine wave reference, capable of serving as a set point for control purposes. This reference can be utilised, for example, as demonstrated in the following video: https://youtu.be/x0tvgowaUfE?si=P_LXpwNw3ohQ54Sz. This reference signal demonstrates one potential application scenario for utilisation within the system.

The “goPosition” function utilises a fundamental PID control mechanism to establish and maintain the desired position within the system. On the other hand, the “goImpedance” function requires three mandatory arguments: the mass, spring, and damper coefficients of the system. An optional argument, the initial position, can also be specified. The default position is set at 125 degrees (2.18166 rad), representing the middle, although any value within the range of 0 to 250 can be utilised as needed.

Similarly, the “goAdmittance” function demands three mandatory arguments: the system’s mass, spring, and damper coefficients. Additionally, two optional arguments are available: the initial position and initial force. The default initial position remains at the midpoint (125 degrees = 2.18166 rad), yet it can be adjusted within the range of 0 to 250. The initial force signifies the force sensitivity of the system and defaults to 0, although it can be modified according to specific requirements.

## 7 Validation and characterisation

In this section, experimental scenarios are considered with the aim of exploiting and validating the design features of the proposed robotic arm. First, a real-time tracking analysis of the joint angle is performed, measuring the difference between the desired and actual position of the joint angle. In particular, two use cases are outlined: the first use case is about impedance control, while the second use case concerns admittance control.

### 7.1 Use case 1: impedance control

**Fig. 14:**
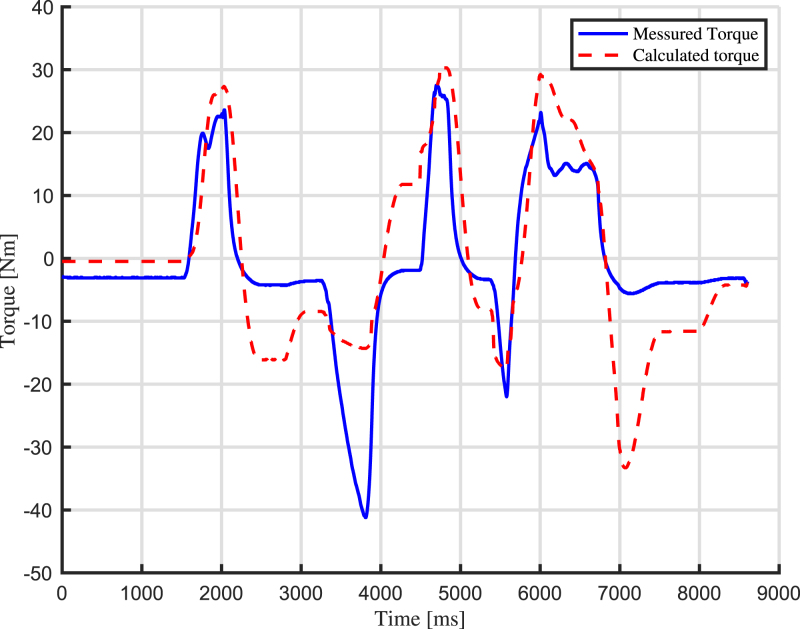
Torque comparison of the arm during the impedance control use case.

**Fig. 15:**
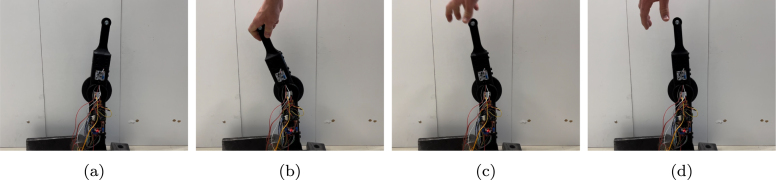
Impedance tests: (a) arm in initial position; (b) force is applied; (c) force is released; (d) to initial position.

The first use case concerns impedance control, which includes monitoring the displacement of the arm and regulating the motor’s torque output correspondingly. The robotic arm’s capability to accurately respond to external forces while maintaining desired levels of compliance is assessed. The results are shown in Fig. 13 and in Fig. 14. The standard deviation between the calculated torque and the actual produced torque is calculated and found to be 9.67 Nm. Through this experiment, the arm’s responsiveness, stability, and overall effectiveness in achieving impedance control is validated. For this test, a video is captured, and the results are shown as pictures of key moments in Fig. 15.

**Fig. 16:**
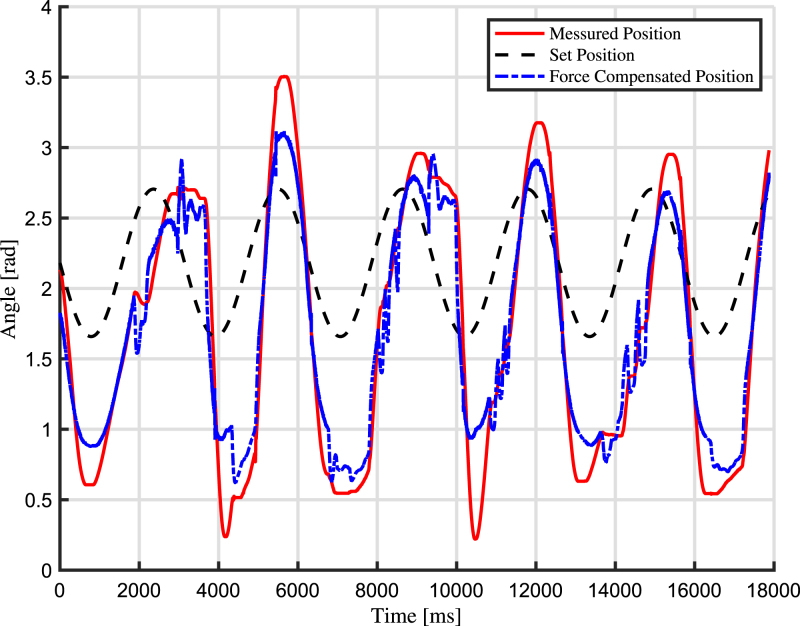
Angle position of the arm during the admittance control use case. (For interpretation of the references to colour in this figure legend, the reader is referred to the web version of this article.)

**Fig. 17:**
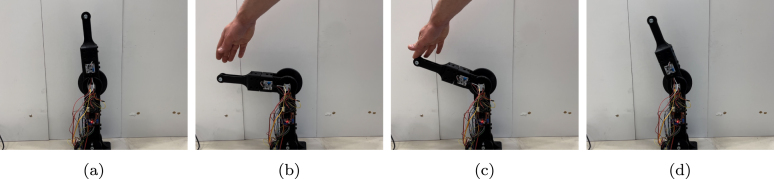
Admittance test: (a) arm in initial position; (b) moving uninterupted; (c) force is applied; (d) returning to path.

### 7.2 Use case 2: admittance control

The second use case is related to admittance control, in which the arm reads the forces in the environment and adapts its own motion correspondingly. The simulations entail testing the arm’s response to a given force profile and evaluating its capacity to track and adjust to external forces in real-time. The results are shown in Fig. 16. The standard deviation between the calculated force compensated angle (blue) and the actual measured actuated angle (red) is calculated and found to be 0.2563 rad. Through this experiment, the arm’s capacity to perform effective admittance control is validated. The actual position of the arm compared to the set point have a considerable overshot. This is due to poor tuning of the PID-controller in addition to imperfection of the spring integration of the arm used for the test. For this test, a video is captured, and the results are shown as pictures of key moments in Fig. 17.

## 8 Conclusions and future works

The adoption of compliant actuators in prosthetics and orthotics is critical to ensuring the user’s safety and comfort. This work introduced an innovative low-cost sensorised elastic actuator design for prosthetics and orthotics that can be customised rapidly and cost-effectively utilising 3D printing technology. This is relevant from an educational perspective. The proposed design is open-source, allowing researchers and practitioners to easily access and modify it. Furthermore, the support for impedance and admittance control techniques increases the system’s adaptability, allowing it to be used in a variety of dynamic circumstances. The project is fully accessible to researchers and practitioners at OSF https://osf.io/4eujq/. Validation results showed a standard deviation of 9.67 Nm between the calculated and measured torque in impedance control, and a standard deviation of 0.2563 rad between the calculated and measured angle in admittance control. These findings demonstrate the design’s effectiveness in adapting to various operational needs in prosthetic and orthotic applications. This research has the potential to help produce low-cost and effective prosthetic and orthotic devices, thereby increasing the quality of life for individuals with disabilities. Further studies can investigate the proposed design’s performance and efficiency in real-world applications, as well as how it might be incorporated into current systems, i.e., exoskeleton for hand rehabilitation [28]. Moreover, novel control algorithms may be implemented [24], [25], [29]. Finally, the possibility of integrating the system with a multi-modal auditory-visual-tactile could be considered [30], [31].

## Ethics statements

The authors confirm that informed consent was obtained from all subjects involved in the validation experiments, in accordance with ethical guidelines.

## CRediT authorship contribution statement

**Filippo Sanfilippo:** Writing – review & editing, Writing – original draft, Supervision, Resources, Project administration, Methodology, Conceptualization. **Martin Økter:** Writing – review & editing, Writing – original draft, Validation, Software, Data curation, Conceptualization. **Jørgen Dale:** Writing – review & editing, Writing – original draft, Investigation, Formal analysis. **Hua Minh Tuan:** Writing – review & editing, Writing – original draft, Visualization, Data curation. **Muhammad Hamza Zafar:** Writing – review & editing, Writing – original draft, Validation, Formal analysis. **Morten Ottestad:** Writing – review & editing, Resources, Investigation.

## Declaration of competing interest

Declarations of interest: None

All authors claim that there is not any conflict of interest regarding the above submission. The work of this submission has not been published previously. It is not under consideration for publication elsewhere. Its publication is approved by all authors and that, if accepted, it will not be published elsewhere in the same form, in English or in any other language, including electronically without the written consent of the copyright-holder.
